# Ochratoxin A Toxicity in Rabbits—A Review

**DOI:** 10.3390/toxins18100415

**Published:** 2026-09-28

**Authors:** Kalina Zhivkova

**Affiliations:** Department of General and Clinical Pathology, Faculty of Veterinary Medicine, Trakia University, Students Campus, 6000 Stara Zagora, Bulgaria; kalina.zhivkova@trakia-uni.bg

**Keywords:** ochratoxin A, rabbits, ochratoxicosis, nephrotoxicity, immunotoxicity, oxidative stress, detoxification, food safety

## Abstract

Ochratoxin A (OTA), a mycotoxin produced by *Aspergillus* and *Penicillium* species, is a widespread feed contaminant with nephrotoxic, hepatotoxic, immunotoxic, reproductive, and teratogenic effects. Despite the particular susceptibility of rabbits to OTA, information on ochratoxicosis in this species remains limited. This review summarizes current evidence on OTA occurrence, toxicokinetics, mechanisms of toxicity, clinicopathological effects, immunotoxicity, and preventive strategies in rabbits. Following gastrointestinal absorption, OTA has a high affinity for plasma proteins and preferentially accumulates in the kidneys and liver. Its toxicity is mediated by the inhibition of protein synthesis, mitochondrial dysfunction, oxidative stress, genotoxicity, apoptosis, and immune dysregulation, ultimately leading to renal and hepatic injury, hematological and biochemical alterations, impaired growth, and reproductive toxicity. OTA-induced immunosuppression may also increase susceptibility to secondary infections, including *Pasteurella multocida*. Importantly, pathological and biochemical alterations may occur following relatively low-level exposure, while residual OTA levels may persist in tissues after exposure is discontinued. Effective prevention of OTA contamination in feed is therefore crucial for controlling ochratoxicosis. Microbial and enzymatic detoxification, probiotics, and phytogenic additives represent promising approaches for the prevention and mitigation of OTA toxicity; however, further studies in rabbits are needed to establish their efficacy and optimal application and to evaluate the effects of different OTA doses, early biomarkers, and tissue residue kinetics.

## 1. Introduction

Mycotoxins are secondary fungal metabolites that represent a major threat to animal health, food safety, and livestock production. Among these compounds, ochratoxin A (OTA) has significant toxicological relevance because of its pronounced nephrotoxic, hepatotoxic, immunotoxic, reproductive, teratogenic, and carcinogenic properties. OTA is widely found in cereals and feedstuffs, and its high chemical stability contributes to its persistence in contaminated feed and increases the risk of transfer through the food chain. Its toxicological significance is further increased by its involvement in mycotoxic nephropathy and its ability to interact with other toxic or infectious factors, thereby increasing disease severity [1,2,3].

The toxic effects of OTA result from several interconnected cellular and molecular mechanisms, including disruption of phenylalanine metabolism, inhibition of protein synthesis, mitochondrial dysfunction, oxidative and nitrosative stress, lipid peroxidation, DNA damage, and induction of apoptosis. These mechanisms particularly affect organs involved in toxin metabolism and elimination, with the kidneys and liver representing the principal target organs. The severity of OTA-induced toxicity depends on the dose and duration of exposure, route of administration, species susceptibility, and individual physiological status. Importantly, impairment of immune function may increase susceptibility to secondary infections and contribute to the progression of clinical and pathological lesions.

Rabbits are considered one of the animal species particularly susceptible to OTA intoxication. Experimental studies have demonstrated that exposure to OTA-contaminated feed may produce a broad spectrum of adverse effects, including reduced feed intake and body weight gain, impaired growth and productivity, renal and hepatic dysfunction, oxidative stress, hematological and biochemical abnormalities, reproductive disturbances, and suppression of both humoral and cell-mediated immune responses. The kidneys are the primary target organs of OTA toxicity in rabbits, particularly the proximal tubular epithelium, which is highly susceptible to degenerative and necrotic changes. Characteristic renal lesions include tubular degeneration and dilation, epithelial necrosis, proteinaceous or hyaline casts, glomerular alterations, interstitial inflammatory infiltration, and, following prolonged or severe exposure, fibrosis and progressive structural changes. Pathological changes have also been reported in the liver, lymphoid organs, heart, reproductive organs, and gastrointestinal tract, indicating that the toxic effects of OTA in rabbits are systemic and not confined to the kidneys.

The toxicokinetic properties of OTA contribute substantially to its toxicity in rabbits. Following oral exposure, OTA is readily absorbed, strongly associated with plasma proteins, and widely distributed throughout the body, with the highest concentrations occurring in the kidneys and liver. Enterohepatic recirculation and prolonged systemic persistence may contribute to prolonged exposure and tissue injury, particularly during continuous intake of contaminated feed. The persistence of OTA in edible tissues represents an important food safety concern because of the potential transfer of the toxin from contaminated feed to rabbit-derived products.

Despite extensive research on OTA in various livestock and laboratory species, information regarding its effects in rabbits remains limited. Available studies demonstrate renal and hepatic injury, oxidative stress, immunosuppression, hematological and biochemical alterations, reproductive and developmental effects, and increased susceptibility to secondary infections. Nevertheless, further research is needed to better define dose-dependent effects, identify early biomarkers, characterize tissue distribution and persistence, and clarify the relationship between oxidative stress and impaired immune function. Particular attention should also be given to the efficacy and safety of preventive approaches. Biological detoxification, probiotics, microbial and enzymatic degradation, and phytogenic additives with antioxidant, hepatoprotective, nephroprotective, and immunomodulatory properties represent promising strategies for mitigating the adverse effects of OTA.

Therefore, this review summarizes and critically evaluates the available evidence on OTA toxicity in rabbits, with particular emphasis on toxicokinetics, mechanisms of action, clinical manifestations, hematological and biochemical alterations, oxidative stress, pathomorphological lesions, immunotoxicity, interactions with infectious diseases, and preventive and detoxification strategies. Understanding the relationship among these effects is important for improving the prevention and management of ochratoxicosis, protecting rabbit health and welfare, maintaining production efficiency, and reducing the risk of OTA entering the food chain.

## 2. Occurrence, Toxicokinetics and Mechanisms of Ochratoxin A Toxicity in Rabbits

Ochratoxin A (OTA) is a widespread mycotoxin produced predominantly by species of *Aspergillus* and *Penicillium* and frequently detected in cereals and animal feeds. Rabbit feeds may also be contaminated with OTA, representing a potential health and food-safety concern given the high susceptibility of rabbits to this toxin. In Argentina, OTA was detected in 25% of examined rabbit feed samples at concentrations of 15–25 ng/g [4,5]. In another survey, mycotoxin-producing fungi were detected in all examined rabbit and chinchilla feed samples, while OTA was identified in 98% of samples at approximately 5 μg/kg [6].

Following oral exposure, OTA is absorbed predominantly in the small intestine, with an estimated absorption of approximately 56% in rabbits [7,8]. After absorption, OTA binds extensively to plasma proteins, particularly albumin, which contributes to its prolonged circulation and sustained tissue exposure. Rabbit-specific pharmacokinetic studies reported a plasma half-life of approximately 8.2 h, an oral bioavailability of 55.6%, and peak plasma concentrations approximately 1 h after oral administration [7]. OTA is excreted primarily in the urine and bile, whereas enterohepatic recirculation may contribute to prolonged systemic exposure. [9].

The kidneys represent the principal target organ and an important site of OTA accumulation in rabbits. The preferential renal exposure may be related, at least in part, to the involvement of organic anion transport systems in the renal handling of OTA and repeated exposure of proximal tubular epithelial cells to the toxin [10]. Importantly, OTA residues have been detected in the kidneys, liver, and skeletal muscle for up to four weeks after exposure, indicating prolonged tissue retention [11]. The highest concentrations have been reported in the kidneys, followed by the liver, mammary gland, and skeletal muscle [12]. Collectively, efficient absorption, extensive plasma protein binding, preferential renal accumulation, and prolonged tissue retention may contribute substantially to the severity of OTA-induced toxicity following repeated dietary exposure.

The molecular mechanisms underlying OTA-induced toxicity are multifaceted and closely interconnected. Due to its structural similarity to phenylalanine, OTA can interfere competitively with phenylalanine-dependent enzymes, particularly phenylalanyl-tRNA synthetase, resulting in impaired protein synthesis [13,14,15,16]. OTA also disrupts DNA and RNA, and mRNA synthesis and affects several cellular metabolic pathways. Furthermore, disruption of mitochondrial transport processes may compromise ATP production and impair cellular energy metabolism [17,18]. These effects are particularly pronounced in tissues with high metabolic demands and may contribute to the increased susceptibility of renal tubular epithelial cells and hepatocytes to OTA-induced injury.

Oxidative stress represents another important component of OTA-induced toxicity. OTA exposure promotes the generation of reactive oxygen species and lipid peroxidation, thereby disrupting cellular redox homeostasis and activating pathways associated with cellular injury and apoptosis. Experimental studies in rabbits have demonstrated increased lipid peroxidation and malondialdehyde (MDA) formation in renal and hepatic tissues following OTA exposure, together with evidence of apoptotic injury [19,20]. The interaction between oxidative stress, mitochondrial dysfunction, and disrupted cellular metabolism may thus contribute to the progressive renal and hepatic injury characteristic of ochratoxicosis.

OTA may also induce genotoxicity and developmental toxicity. Experimental evidence indicates that OTA exposure can induce DNA strand breaks and chromosomal alterations and interfere with cellular replication processes, potentially contributing to genomic instability [21,22,23]. In rabbits, maternal exposure has been associated with embryotoxicity and congenital abnormalities, including skeletal defects, hydrocephalus, microphthalmia, and renal agenesis, with adverse effects becoming more pronounced at higher exposure levels [24,25,26].

Overall, the toxicokinetic profile of OTA in rabbits is characterized by efficient gastrointestinal absorption, extensive plasma protein binding, preferential accumulation in the kidneys and liver, and prolonged tissue retention. These toxicokinetic properties interact with multiple molecular mechanisms, including inhibition of protein synthesis, mitochondrial dysfunction, oxidative stress, apoptosis, and genotoxicity. The resulting cellular injury provides a mechanistic basis for the renal, hepatic, hematological, immunological, reproductive, and developmental alterations described in the subsequent sections. It should provide a concise and precise description of the experimental results, their interpretation, and the experimental conclusions that can be drawn.

## 3. Toxicopathological Effects of Ochratoxin A in Rabbits

Rabbits are highly susceptible to OTA, showing greater sensitivity than several commonly used laboratory species, including rats, mice, and guinea pigs [27]. OTA toxicity in rabbits is characterized by a broad spectrum of systemic effects, including nephrotoxicity, hepatotoxicity, immunotoxicity, hematological and metabolic disturbances, reproductive and developmental toxicity, and increased susceptibility to secondary infections. The reported LD_50_ is approximately 10 mg/kg body weight [28], while teratogenic effects have been demonstrated at substantially lower exposure levels [24]. Despite this high susceptibility and the documented occurrence of OTA in rabbit feed, information on naturally occurring ochratoxicosis in this species remains relatively limited [29].

Experimental evidence indicates that prolonged dietary exposure to OTA causes progressive systemic toxicity, which may remain clinically inapparent until substantial tissue injury has occurred. The kidneys are the primary target organs; however, the liver, immune system, gastrointestinal tract, myocardium, reproductive organs, and central nervous system may also be affected. Lesion severity is determined predominantly by the dose and duration of exposure and may be further modulated by the animal’s physiological status, concurrent exposure to other mycotoxins, and secondary infections.

### 3.1. Clinical Signs

The clinical manifestations of ochratoxicosis are generally nonspecific and depend on the dose, duration of exposure, age, and physiological status of the animals. Chronic exposure frequently follows a predominantly subclinical course, with overt signs becoming apparent only after considerable toxin accumulation and organ injury. Reduced feed intake, anorexia, impaired feed utilization, impaired growth, and decreased body weight gain are among the most consistently reported manifestations. Feed consumption may decrease by approximately 20–60%, resulting in reduced productive performance, particularly in growing and fattening rabbits. More pronounced intoxication in animals may be accompanied by lethargy, depression, dehydration, gastrointestinal disturbances, respiratory signs, and increased susceptibility to secondary infections [30] (Table 1). Importantly, impaired growth performance has also been reported following relatively low dietary exposure, including 19 μg OTA/kg feed and 0.03 mg/kg feed [31,32] (Table 1). Prolonged high-dose exposure may additionally alter organ weights. Male rabbits receiving 10 mg OTA/kg feed for 90 days developed apathy, lethargy, respiratory disturbances, hair loss, and reduced feed intake, accompanied by increased absolute and relative weights of the liver, kidneys, and spleen and decreased absolute and relative weights of the heart and lungs [33] (Table 1).

Reproductive and developmental toxicity is another important component of the clinical profile. Teratogenic effects have been reported at oral doses as low as 0.050 mg/kg body weight, with increasing OTA doses associated with more pronounced embryo resorption and increased frequency of visceral and skeletal abnormalities [24,25,26]. Overall, the nonspecific clinical presentation emphasizes the limited diagnostic value of clinical signs alone and the need to integrate them with biochemical, pathological, and toxicological findings.

### 3.2. Hematological and Biochemical Changes

OTA intoxication in rabbits produces a characteristic biochemical profile reflecting renal, hepatic, metabolic, and hematological injury. Among the most consistent abnormalities are increased serum urea and creatinine, elevated hepatic enzyme activities, decreased total protein and albumin, and disturbances in glucose, lipid, and electrolyte metabolism.

Progressive reductions in serum total protein and albumin have been repeatedly demonstrated in rabbits exposed to OTA. Mir and Dwivedi [34] observed decreasing concentrations in rabbits receiving 1000 or 2000 µg OTA/kg feed, while similar findings were reported following exposure to 1–2 mg/kg feed and 0.3 mg/kg feed [35,36] (Table 1). Hypoproteinemia and hypoalbuminemia are potential consequences of a combination of impaired hepatic protein synthesis, renal protein loss, reduced feed intake, and gastrointestinal dysfunction [38]. Reduced serum chloride has also been described during prolonged exposure [34,35] (Table 1).

Alterations in glucose homeostasis are less consistent. Dose- and time-dependent hypoglycemia has been described and has been associated with neurological manifestations in severe ochratoxicosis [34,39] (Table 1). Conversely, increased serum glucose has been reported under other experimental conditions [36], suggesting that glucose responses may depend on the exposure regimen and metabolic status of the animals.

The most reliable biochemical indicators of OTA-induced nephrotoxicity are increased serum urea and creatinine (Table 1). Significant elevations have been reported following exposure to 1000–2000 µg OTA/kg feed and may appear within the first weeks of intoxication [34]. Similar changes have been demonstrated at 1–2 mg/kg feed, 0.03–0.06 mg/kg body weight in pregnant rabbits, and 0.3 mg/kg feed [26,35,36] (Table 1). Because both parameters depend strongly on renal filtration, their elevation is consistent with progressive nephron dysfunction and may precede severe clinical disease.

Hepatic injury is evidenced by increased serum activities of Alanine aminotransferase (ALT), Aspartate aminotransferase (AST), and Alkaline phosphatase (ALP) (Table 1). Elevated ALT and AST have been observed following exposure to 1000–2000 µg OTA/kg feed, with changes detectable early during intoxication [34]. Similar increases have been reported at 19 μg/kg feed, 0.03–0.06 mg/kg body weight, and 0.2–0.3 mg/kg feed [26,31,36,37] (Table 1). Increased ALP has also been documented and appears to depend on both OTA dose and exposure duration.

Additional systemic abnormalities include increased triglyceride and cholesterol concentrations, increased Lactate dehydrogenase (LDH) activity, and reduced serum sodium and potassium [31,36]. These changes indicate that OTA toxicity extends beyond the kidneys and liver, affecting overall cellular and metabolic homeostasis.

Hematological alterations include decreased erythrocyte counts, hemoglobin concentrations, and packed cell volume, consistent with the development of anemia and tissue hypoxia [26]. Leukocyte abnormalities are variable but may include leukocytosis, heterophilia, monocytosis, lymphopenia, and eosinopenia [36] (Table 1). High and prolonged OTA exposure has also been associated with thrombocytopenia and prolonged coagulation time [40]. Collectively, these findings indicate that serum urea, creatinine, ALT, AST, ALP, total protein, albumin, and selected hematological variables may provide useful complementary indicators for the early detection and monitoring of OTA-induced systemic toxicity (Table 1).

### 3.3. Oxidative Stress, Lipid Peroxidation, and Renal Injury

Oxidative stress represents a central mechanism of OTA-induced toxicity in rabbits. Dietary exposure promotes excessive generation of reactive oxygen species (ROS), lipid peroxidation, protein oxidation, and disruption of endogenous antioxidant defenses. In rabbits exposed to 1 mg OTA/kg feed, increased MDA concentrations and alterations in catalase (CAT) and superoxide dismutase (SOD) activities were detected in renal and hepatic tissues after 30 and 60 days [41]. Increased serum MDA and nitric oxide (NO), together with decreased total antioxidant capacity and CAT activity, have also been reported at 0.3 mg/kg OTA [36]. Importantly, increased MDA has been observed even at 19 μg/kg feed [31], indicating that oxidative imbalance may occur at relatively low dietary exposure levels.

OTA-induced oxidative injury affects not only membrane lipids but also proteins, DNA, and extracellular matrix remodeling. Increased protein carbonyl formation indicates irreversible oxidative modification of cellular proteins, whereas increased 8-hydroxy-2′-deoxyguanosine (8-OHdG) reflects oxidative DNA damage. Oxidative injury has also been associated with increased hydroxyproline and Pro-collagen I alpha 1 (COL1A1) expression, linking chronic oxidative stress with collagen deposition and fibrotic remodeling [44,45].

Recent experimental evidence further supports this proposed mechanism. OTA-exposed rabbits showed increased ROS, NO, ascorbate radicals, protein oxidation, protein carbonyls, 8-OHdG, MDA, hydroxyproline, advanced glycation end products, Tumor necrosis factor alpha (TNF-α), COL1A1, and Kidney Injury Molecule-1 (KIM-1), together with reduced SOD and glutathione peroxidase-1 (GPx-1) activity [42]. KIM-1 is particularly relevant because its increased expression provides direct evidence of proximal tubular epithelial injury [43].

Collectively, these findings support a mechanistic sequence in which OTA-induced ROS and reactive nitrogen species initiate lipid, protein, and DNA oxidation, compromise mitochondrial and cellular function, activate inflammatory signaling, and promote progressive tubular injury and fibrotic remodeling. Oxidative and nitrosative stress therefore represent important links between molecular OTA toxicity and the structural renal and hepatic lesions observed during chronic ochratoxicosis.

### 3.4. Pathomorphological Changes Associated with Ochratoxicosis

The kidneys are the primary target organ of OTA toxicity in rabbits, and proximal tubular degeneration represents the most consistent histopathological lesion. Macroscopically, OTA-exposed rabbits may show enlarged, pale, soft, and mottled kidneys, with the severity of changes increasing with prolonged exposure [41].

Histologically, renal lesions include granular and vacuolar degeneration of proximal tubular epithelial cells, epithelial desquamation and disintegration, tubular dilation, hyaline or cellular casts, vascular congestion, interstitial mononuclear infiltration, and glomerular alterations. Rabbits receiving 1 mg OTA/kg feed for 30–60 days developed progressive proximal tubular degeneration together with tubular casts, haemorrhage, and glomerular changes [41]. Ultrastructural investigations have demonstrated mitochondrial membrane damage, dilation of the endoplasmic reticulum, thickening of the glomerular basement membrane, and endothelial degeneration [41,46] (Table 2).

Comparable lesions have been observed at lower exposure levels. Rabbits fed 0.3 mg OTA/kg feed developed marked tubular vacuolar degeneration, cystic tubular dilation, compression atrophy, mononuclear infiltration, hyaline casts, and focal necrosis [36]. Glomerular necrosis and tubular alterations have also been reported at 0.03–0.06 mg/kg body weight [26], while tubular degeneration and necrosis have been described at 19 μg/kg feed [31].

Severe prolonged exposure may progress to tubular atrophy, interstitial fibrosis, and glomerular hyalinization [33,43] (Table 2). Thus, OTA-induced renal pathology follows a progressive pattern from proximal tubular degeneration to irreversible structural remodeling.

The liver is another major target organ of OTA toxicity. Hepatic lesions include granular and vacuolar degeneration, cytoplasmic vacuolation, fatty change, sinusoidal and central venous congestion, focal necrosis, biliary epithelial proliferation, and peribiliary fibrosis. Rabbits exposed to 10 mg/kg feed for 90 days developed severe disruption of hepatic architecture, focal necrosis, fatty and hyaline degeneration, and hepatocellular necrosis [33]. Similar but generally milder hepatic alterations have been reported following exposure to substantially lower OTA doses, including 19 μg/kg feed, 0.06 mg/kg body weight, and 0.2 mg/kg body weight [26,31,37] (Table 2).

Recent studies confirm that OTA-induced pathology in rabbits is multisystemic. Degenerative lesions have been identified not only in the kidneys and liver but also in the myocardium, lungs, spleen, intestine, brain, cerebellum, testes, and ovaries [42]. Cardiac lesions may include myofibrillar degeneration, fatty vacuolation, myolysis, and necrosis [36]. Testicular alterations include degeneration of the spermatogenic epithelium, intertubular oedema, seminiferous tubular disruption, depletion of spermatozoa, and increased interstitial connective tissue [41] (Table 2).

Lymphoid organs are particularly relevant because structural injury corresponds closely with OTA-induced immunosuppression. Degeneration and necrosis of lymphoid cells, depletion of white pulp, thymic hypocellularity, haemorrhage, and oedema have been reported in the spleen, thymus, and lymph nodes of OTA-exposed rabbits [35]. Gastrointestinal lesions include lymphocytic infiltration and epithelial desquamation, which may contribute to impaired nutrient absorption and reduced growth [26,31] (Table 2).

The developmental effects of OTA are also reflected morphologically. Pregnant rabbits exposed to 0.025–0.100 mg/kg body weight showed dose-dependent reductions in fetal viability, weight, and length, together with musculoskeletal defects, hydrocephalus, microphthalmia, and renal agenesis [24]. Severe histopathological alterations have additionally been documented in fetal liver, kidney, brain, heart, and eye tissues [25].

Recent studies in rabbits have also provided evidence of central nervous system involvement. Mild neuronal pyknosis and lysis, pericellular and pericapillary oedema, microglial proliferation, and lymphocytic infiltration have been observed in the brain, together with oedema and degenerative changes in Purkinje cells and the cerebellar white matter [42]. Although less prominent than renal and hepatic pathology, these lesions support the concept that neurotoxicity contributes to the systemic toxicological profile of OTA in rabbits.

Overall, the pathomorphological pattern of ochratoxicosis is characterized predominantly by renal tubular injury, accompanied by significant extrarenal lesions. The occurrence of comparable pathological alterations across a broad range of OTA concentrations supports the high susceptibility of rabbits and the value of histopathology as an important endpoint in the assessment of OTA toxicity.

### 3.5. Immunosuppressive Effects of Ochratoxin A

OTA exerts pronounced immunotoxic effects in rabbits, affecting both humoral and cell-mediated immunity. The immunosuppressive phenotype is characterized by lymphoid tissue degeneration, alterations in leukocyte populations, impaired lymphocyte proliferation, reduced antibody responses, defective phagocytic activity, and dysregulated cytokine signaling. These effects are largely attributed to the inhibition of protein synthesis, oxidative and mitochondrial damage, and the induction of apoptosis and necrosis in immunocompetent cells.

Reduction of circulating lymphocytes and depletion of immunocompetent cells in the spleen, thymus, mesenteric lymph nodes, and submucosal lymphoid tissues have been repeatedly reported in OTA-intoxicated rabbits [40,51]. OTA-induced inhibition of protein synthesis may impair lymphocyte proliferation and differentiation, while apoptosis and necrosis further reduce the population of antibody-producing and effector cells [52,53].

Functional studies confirm impairment of both cellular and humoral immune responses. Rabbits exposed to 0.75 mg OTA/kg feed for 60 days exhibited reduced delayed-type hypersensitivity responses, decreased lymphocyte proliferation, and altered antibody responses to sheep red blood cells [48]. Reduced lymphocyte transformation and antibody production have also been reported in other experimental studies [41,54].

Cytokine dysregulation represents an additional component of OTA immunotoxicity. Increased Interleukin-2 (IL-2), Interleukin-6 (IL-6), and TNF-α concentrations have been associated with decreased phagocytic activity and reduced lymphocyte stimulation index in rabbits [49]. More recent investigations demonstrated increased IL-6, IL-1β, (Interferon-gamma) IFN-γ, and TNF-α in the liver, kidneys, blood, and pancreas, linking inflammatory signaling with oxidative stress and organ injury [42].

Particularly relevant evidence has emerged from vaccine-response studies. Rabbits exposed to 2 mg OTA/kg feed and immunized against rabbit hemorrhagic disease virus (RHDV) exhibited a markedly impaired antibody response, accompanied by lymphoid depletion and degenerative lesions in the spleen and thymus. [47]. These findings demonstrate that OTA-induced immunotoxicity is functionally relevant and may compromise the development of protective vaccine-induced humoral immunity.

Thus, OTA-induced immunosuppression in rabbits is a multifactorial process involving lymphoid cell depletion, inhibition of immune-cell proliferation, altered cytokine signaling, impaired antibody production, and reduced cellular immune response. These effects substantially impair host defense mechanisms and provide a biological basis for the increased susceptibility of OTA-exposed rabbits to secondary infections.

### 3.6. Interaction of OTA with Infectious Diseases

The immunosuppressive effect of OTA is of particular practical significance, as it increases susceptibility to opportunistic and secondary bacterial infections. Experimental evidence demonstrates that combined exposure to OTA and *Pasteurella multocida* produces more severe clinical and pathological effects than either factor alone.

Rabbits exposed to 1000 or 2000 µg OTA/kg feed and subsequently challenged with *Pasteurella multocida* developed more pronounced depression, weakness, dehydration, reduced feed intake and body weight gain, respiratory distress, and higher mortality than rabbits exposed only to the bacterium [50]. OTA-exposed rabbits also exhibited increased bacterial loads in the respiratory tract, particularly at 2000 µg/kg, supporting the conclusion that OTA-induced immunosuppression impairs host resistance to infection.

Recent experimental findings provide additional evidence for this interaction. In growing rabbits exposed to 2 mg OTA/kg feed for 80 days, suppression of the humoral immune response was associated with secondary pasteurellosis and mortality [47]. Rabbits that died from secondary infection developed severe pulmonary lesions, including catarrhal or purulent bronchopneumonia, croupous pneumonia, haemorrhage, pleuritis, and marked pulmonary oedema. *Pasteurella cuniculiseptica* was detected in affected lungs, while the most severe respiratory lesions were associated with secondary bacterial infection rather than OTA exposure alone.

These observations indicate that the toxicological significance of OTA extends beyond direct renal and hepatic injury. By suppressing humoral and cellular immune responses and altering inflammatory and lymphoid function, OTA may act as an important predisposing factor for infectious disease and increase disease-associated mortality. This interaction is particularly relevant under practical production conditions, where chronic dietary exposure to OTA may occur concurrently with environmental stress and opportunistic pathogens.

## 4. Risk Assessment, Hygienic Measures, and Diagnosis of Ochratoxicosis in Rabbits

The widespread occurrence and toxicological effects of OTA make contamination of cereals and cereal-based feeds an important concern for rabbit health, production efficiency, and food safety. In the European Union, Commission Recommendation (EU) 2026/1801 establishes a guidance value of 0.05 mg OTA/kg for cereals and cereal products and a specific guidance value of 0.01 mg OTA/kg for complete feed for rabbits [55]. In its risk assessment, the EFSA CONTAM Panel also identified 0.01 mg OTA/kg complete feed as the reference point (RP) for adverse health effects in rabbits [56]. Although these values are the same, the EU guidance value applies to feed, whereas the EFSA RP was established for the assessment of adverse health effects in rabbits. These recommendations emphasize the importance of controlling OTA contamination and minimizing dietary exposure in rabbit production.

The food safety relevance of OTA is further supported by its transfer from contaminated feed to animal tissues. In rabbits exposed to OTA-contaminated diets, residues have been detected predominantly in the kidneys and liver, but also in mammary tissue and skeletal muscle. In lactating rabbits receiving 10–20 g/kg body weight/day, higher OTA transfer was observed in the liver than in the mammary gland and muscle. [12]. Rabbits’ characteristic cecotrophic behavior may also contribute to repeated exposure when OTA is present in cecotropes. Consequently, effective risk management should focus primarily on preventing fungal growth and OTA formation through appropriate feed ingredient selection, adequate drying, temperature and moisture control, hygienic storage, and regular monitoring of feed batches.

Because the clinical manifestations of ochratoxicosis are generally nonspecific, reliable diagnosis requires integration of clinical, pathological, biochemical, and toxicological findings. OTA can be determined in feed, serum, and tissue samples using conventional chromatographic methods such as TLC, HPLC, and HPLC-FLD, while LC-MS/MS provides higher analytical sensitivity and specificity. ELISA and other immunoassays remain useful for screening because of their accessibility and relative simplicity [57,58,59,60]. More recent antibody-based assays, aptasensors, and nanomaterial-based platforms may further facilitate rapid OTA detection [61,62,63], whereas PCR-based methods can support the identification of toxigenic fungal species in contaminated feed [64].

Pathomorphological examination remains an important component of diagnosis. Enlarged, pale, or mottled kidneys together with degeneration and necrosis of proximal tubular epithelial cells are highly suggestive of OTA-associated nephropathy. However, these lesions are not entirely specific and should be interpreted together with biochemical evidence of renal and hepatic dysfunction and direct OTA determination in feed or biological samples [65,66,67,68]. When OTA is no longer detectable, information on feed-storage conditions, evidence of fungal contamination, increased prevalence of renal lesions at slaughter, negative microbiological findings, and the clinical history may provide additional diagnostic support.

Overall, prevention and early detection remain the most effective strategies for controlling ochratoxicosis in rabbits. An integrated approach combining feed hygiene, routine OTA monitoring, biochemical assessment, pathological examination, and confirmatory toxicological analysis is essential for reducing animal-health risks, limiting production losses, and minimizing OTA transfer into the food chain.

## 5. Preventive and Detoxification Strategies Against OTA Toxicity in Rabbits

Prevention of OTA contamination remains the most effective strategy for controlling ochratoxicosis because of the high chemical stability of OTA, its efficient gastrointestinal absorption, and persistence in target tissues. Appropriate harvesting and drying practices, control of moisture and temperature during storage, prevention of fungal growth, and routine monitoring of feed ingredients therefore constitute the first line of defense. Once contamination has occurred, additional strategies may be required to reduce OTA bioavailability and mitigate its toxic effects.

Chemical decontamination may reduce mycotoxin concentrations under specific conditions; however, its practical application is limited by possible deterioration of the nutritional and technological properties of feed and by concerns regarding chemical residues [69,70,71]. Similarly, commonly used mineral adsorbents, including kaolin, sepiolite, and bentonite, generally show limited efficacy against OTA and may also bind essential nutrients [72,73,74,75]. These limitations have stimulated increasing interest in biological detoxification and dietary protective strategies.

The use of microorganisms and microbial enzymes represents a promising approach to OTA detoxification through adsorption, degradation, or biotransformation. Several bacterial, fungal, and yeast species have demonstrated ability to mitigate OTA-induced toxicity, while hydrolysis of the OTA amide bond by enzymes such as carboxypeptidases, proteases, lipases, and ochratoxinase may generate less toxic metabolites [76,77,78,79,80,81]. Probiotic preparations may additionally reduce intestinal toxin bioavailability and contribute to the maintenance of gastrointestinal and immune homeostasis [82].

Studies in rabbits provide evidence that selected biological and dietary additives can attenuate OTA-induced toxicity. Supplementation with humic acid, *Bacillus subtilis* and *B. licheniformis* (BioPlus 2B), or mannan oligosaccharides (Bio-Mos) improved growth-related parameters and reduced mortality, hepatic and renal dysfunction, pathological lesions, and oxidative alterations in OTA-exposed rabbits [31]. Dietary *Aspergillus awamori* also attenuated OTA-induced growth depression, hematological and biochemical alterations, immune dysfunction, oxidative stress, and renal and cardiac lesions [36]. Selenium supplementation reduced OTA-associated immunosuppressive and cytotoxic effects [49], while selenomethionine (0.4 mg/kg) attenuated adverse effects induced by OTA exposure at 0.2 mg/kg body weight in rabbits [37]. These findings indicate that biological and antioxidant-based strategies may act both by limiting toxin bioavailability and by strengthening host defense mechanisms.

Phytogenic additives are of particular interest as protective strategies against OTA-toxicosis because their antioxidant, anti-inflammatory, hepatoprotective, nephroprotective, and immunomodulatory activities may counteract several mechanisms involved in OTA toxicity. Among relevant natural agents are *Silybum marianum*, silymarin, *Withania somnifera* (ashwagandha), and *Centella asiatica* (Gotu kola). Experimental studies in different animal species support the protective potential of these phytogenic additives [83,84,85] (Table 3). Importantly, direct evidence is also available in rabbits. In an 80-day experimental study, dietary exposure to 2 mg OTA/kg feed reduced body weight, suppressed the humoral immune response, and caused pronounced pathological alterations, particularly in the kidneys, liver, and spleen. Supplementation with *Silybum marianum*, silymarin, *Withania somnifera*, or *Centella asiatica* reduced the severity of OTA-induced lesions, although the magnitude of protection differed among the preparations [47] (Table 3).

Among the investigated phytogenic interventions, silymarin and *Withania somnifera* provided the most pronounced protection against OTA-induced suppression of the humoral immune response, while all four preparations reduced the severity of renal and hepatic lesions [47]. Nevertheless, secondary pasteurellosis and mortality were still observed in some supplemented groups, demonstrating that phytogenic supplementation may attenuate, but does not completely prevent, OTA-associated immunosuppression and increased susceptibility to infection. These strategies should therefore be considered complementary protective measures rather than substitutes for effective control of feed contamination.

Other natural compounds may also have protective potential. Hydroxytyrosol, a phenolic antioxidant present in olive oil, attenuated OTA-induced oxidative stress and renal dysfunction in cellular models [86]. Chamomile oil has been associated with improved feed intake in rabbits and protection against OTA toxicity in other animal species [87,88]. However, direct evidence demonstrating their efficacy against OTA toxicity in rabbits remains insufficient.

Overall, biological detoxification, probiotics, antioxidant micronutrients, and phytogenic additives represent promising complementary approaches for mitigating OTA toxicity in rabbits. Their potential benefits include reduction of toxin bioavailability, degradation or biotransformation of OTA, reinforcement of antioxidant defenses, preservation of renal and hepatic function, and modulation of immune responses. However, prevention of feed contamination should remain the primary control strategy. Current evidence for most protective interventions remains predominantly experimental, with considerable variation in dose, formulation, duration of exposure, and evaluated outcomes. Future rabbit studies should therefore use standardized in vivo designs and environmentally relevant OTA concentrations and should integrate productive performance, OTA bioavailability and tissue residues, renal and hepatic biomarkers, oxidative stress, immune function, and histopathological endpoints. Such investigations are necessary to establish effective doses, safety, and practical applicability before these interventions can be recommended for routine use in rabbit production.

## 6. Discussion

Ochratoxin A represents a particularly relevant mycotoxicological hazard in rabbits because the susceptibility of this species is combined with the widespread occurrence of the toxin in cereal-based feeds. The available evidence demonstrates that ochratoxicosis in rabbits should be regarded as a systemic toxicological disorder rather than exclusively as a renal disease. Although the kidneys remain the principal target organ, hepatic, hematological, immunological, reproductive, gastrointestinal, and developmental alterations have also been consistently reported. Importantly, significant biochemical and pathological alterations may occur during chronic or relatively low-level OTA exposure without prominent clinical manifestations, indicating that the absence of overt clinical disease does not necessarily imply the absence of toxic injury.

The pronounced susceptibility of rabbits appears to be closely related to the toxicokinetic behavior of OTA. Following oral exposure, the toxin is efficiently absorbed, extensively bound to plasma proteins, and distributed to several tissues, with preferential accumulation in the kidneys and liver. Enterohepatic recirculation and persistence of OTA residues after cessation of exposure may further prolong target-organ exposure. Consequently, repeated dietary intake may produce cumulative toxicity even when clinical signs remain nonspecific. This observation is particularly relevant under practical feeding conditions, where chronic exposure to low or fluctuating OTA concentrations is more likely to occur than acute intoxication.

Renal toxicity represents the most consistent manifestation of ochratoxicosis in rabbits. Increased serum urea and creatinine concentrations are consistent with structural renal lesions, including proximal tubular degeneration and necrosis, tubular dilation and atrophy, glomerular damage, vascular changes, and interstitial inflammatory responses. Increased expression of renal injury markers, such as KIM-1, further supports proximal tubular epithelial damage as a central feature of OTA nephrotoxicity. Collectively, these findings demonstrate the close relationship between functional biomarkers and histopathological lesions and suggest that sensitive renal biomarkers may facilitate the detection of OTA-induced renal injury before advanced clinical disease develops.

Hepatic involvement is also an important component of systemic OTA toxicity. Increased serum levels of AST, ALT, and ALP, together with hepatocellular degeneration, necrosis, vacuolation, fatty changes, vascular congestion, and fibrosis, indicate substantial hepatic injury. Reduced serum total protein and albumin concentrations should therefore be interpreted as multifactorial changes associated with impaired hepatic protein synthesis, renal protein loss, reduced feed intake, and gastrointestinal dysfunction rather than as isolated biochemical abnormalities. The concurrent involvement of the kidneys and liver is consistent with the distribution, metabolism, and elimination of OTA and further emphasizes the systemic nature of chronic OTA exposure.

Oxidative stress represents an important connection between OTA exposure and organ injury. Increased lipid peroxidation and MDA concentrations, together with alterations in CAT, SOD, total antioxidant capacity, and other components of the antioxidant defense system, indicate disruption of cellular redox balance. These changes, together with mitochondrial dysfunction, impaired ATP production, protein oxidation, inflammatory signaling, and apoptosis, may further contribute to renal and hepatic injury. The occurrence of oxidative disturbances at comparatively low OTA exposure levels is particularly relevant, as it suggests that oxidative stress may precede advanced structural damage and could potentially serve as an early indicator of toxicity.

Another major finding emerging from studies in rabbits is the importance of OTA-induced immunotoxicity. OTA affects both humoral and cell-mediated immunity through inhibition of protein synthesis, impaired proliferation of immunocompetent cells, apoptosis and necrosis of lymphoid cells, altered cytokine signaling, and lymphoid tissue depletion. Changes in lymphocyte populations, suppressed antibody responses and lymphocyte proliferation, and alterations in IL-2, IL-6, and TNF-α indicate that multiple levels of immune regulation may be affected simultaneously. Thus, immunosuppression should be regarded as an important toxicological consequence of OTA exposure rather than merely a secondary manifestation of systemic toxicity.

This interpretation is supported by experimental evidence linking OTA-induced immunosuppression to increased susceptibility to infectious diseases. Rabbits exposed to OTA and subsequently challenged with *Pasteurella multocida* develop more severe clinical and pathological manifestations, higher bacterial loads, and increased mortality compared with non-intoxicated animals. Further evidence indicates that dietary exposure to 2 mg OTA/kg feed can impair the humoral immune response and may predispose rabbits to secondary pasteurellosis, accompanied by severe respiratory lesions and mortality. Collectively, these findings support a biological link between feedborne OTA exposure, impaired host defense, and increased susceptibility to secondary infections. Accordingly, the adverse effects of OTA under field conditions may result not only from its direct nephrotoxic and hepatotoxic effects but also from increased susceptibility to opportunistic and secondary pathogens.

Reproductive and developmental toxicity further contributes to the toxicological significance of OTA in rabbits. Embryotoxicity, reduced fetal growth, embryo resorption, and skeletal and visceral abnormalities have been reported following maternal OTA exposure. These effects may be associated with the ability of OTA to interfere with protein synthesis, mitochondrial function, redox homeostasis, and cellular proliferation. However, compared with renal and hepatic toxicity, evidence regarding reproductive and developmental effects and their long-term consequences for offspring remains limited and warrants further investigation.

A major limitation of the current evidence is the considerable heterogeneity among experimental studies. Differences in OTA dose, duration and route of exposure, age, sex, and physiological status of the animals, feed composition, and evaluated endpoints complicate direct comparisons and hinder the establishment of clear dose–response relationships. Furthermore, evidence on naturally occurring ochratoxicosis in commercial rabbit production remains scarce, despite the documented occurrence of OTA in rabbit feeds. The toxicological significance of prolonged low-dose exposure therefore deserves particular attention, especially since biochemical, oxidative, hematological, and pathological alterations have been reported at OTA concentrations substantially lower than those associated with overt clinical intoxication.

The persistence of OTA in rabbit tissues also has important implications for food safety. Detection of OTA in the kidneys, liver, edible tissues, and skeletal muscle indicates that exposure is relevant not only to animal health but also to food safety. Although tissue distribution is strongly organ-dependent, the potential occurrence of OTA residues in edible tissues emphasizes the importance of preventing exposure at the feed level rather than relying on clinical detection of affected animals. Appropriate feed production and storage practices, moisture control, monitoring of susceptible feed ingredients, and sensitive analytical methods should therefore constitute the primary measures for controlling OTA exposure and reducing the risk of ochratoxicosis.

Once feed has been contaminated, effective detoxification remains challenging. Conventional nonspecific mineral binders may have limited efficacy against OTA and may also interact with nutrients. Biological strategies based on microorganisms, probiotics, and OTA-degrading enzymes therefore represent promising alternatives. Phytogenic compounds are also of particular interest because their antioxidant, hepatoprotective, nephroprotective, anti-inflammatory, and immunomodulatory properties may simultaneously counteract several mechanisms involved in OTA-induced toxicity.

Among the phytogenic agents evaluated or proposed, *Silybum marianum* and its active complex silymarin, *Withania somnifera*, and *Centella asiatica* represent promising protective agents. Experimental evidence indicates that these compounds may attenuate oxidative stress, preserve hepatic and renal function, and support immune responses. Of particular relevance, recent studies in rabbits indicate that silymarin and *Withania somnifera* may partially preserve the humoral immune response during OTA exposure. This finding suggests that protective strategies may reduce not only direct organ injury but also some of the secondary consequences associated with OTA-induced immunosuppression.

Nevertheless, evidence supporting the protective effects of phytogenic agents against OTA toxicity specifically in rabbits remains limited. Findings obtained in poultry, rodents, other animal species, or experimental models involving different toxicants cannot be directly extrapolated to rabbit ochratoxicosis. Further studies should therefore determine effective doses, bioavailability, long-term safety, mechanisms of action, and potential effects on OTA toxicokinetics and tissue residues. Importantly, improvement in individual biochemical or oxidative stress parameters should not alone be interpreted as evidence of successful detoxification. Protective efficacy should ideally be supported by concurrent improvements in clinical performance, organ function, immune competence, and histopathological lesions.

Taken together, current evidence supports a multifactorial model of OTA toxicity in rabbits, in which toxicokinetic persistence contributes to prolonged target-organ exposure, while oxidative stress, mitochondrial dysfunction, inhibition of protein synthesis, apoptosis, and immune dysregulation collectively contribute to progressive systemic injury. The resulting toxicological response is influenced not only by OTA dose and duration of exposure but also by the physiological status of the animal and concurrent nutritional, environmental, and infectious stressors. This framework may help explain the considerable variability in the clinical expression of ochratoxicosis and highlights the importance of evaluating OTA toxicity within the broader biological context of rabbit production.

## 7. Conclusions

Ochratoxin A represents a significant toxicological hazard in rabbits because of the high susceptibility of this species and the potential occurrence of OTA in cereal-based feeds. Available evidence indicates that OTA toxicity is systemic and involves interconnected nephrotoxic, hepatotoxic, immunotoxic, oxidative, reproductive, and developmental effects. The kidneys are the primary target organ; however, hepatic injury, impaired growth, hematological alterations, oxidative stress, immune dysfunction, and reproductive toxicity also contribute substantially to the overall toxicological burden.

Of particular importance is the evidence that OTA-induced immunosuppression may increase susceptibility to secondary bacterial infections, including those caused by Pasteurella multocida, thereby aggravating pathological lesions and potentially contributing to mortality. This interaction extends the toxicological significance of OTA beyond its direct nephrotoxic and hepatotoxic effects and highlights immune dysfunction as an important component of ochratoxicosis in rabbits.

The occurrence of toxicological alterations during prolonged exposure to relatively low OTA concentrations, together with its persistence in tissues, indicates that risk assessment should not rely solely on overt clinical signs. Prevention of feed contamination, appropriate storage and feed-quality management, and regular monitoring of OTA levels should therefore remain the primary strategies for controlling exposure, reducing the risk of ochratoxicosis, and limiting OTA residues in rabbit-derived food products.

Biological detoxification and phytogenic protective strategies represent promising complementary approaches. In particular, silymarin, *Silybum marianum*, *Withania somnifera*, and *Centella asiatica* warrant further investigation because of their antioxidant, hepatoprotective, nephroprotective, and immunomodulatory properties. However, evidence specifically demonstrating their protective efficacy against OTA-induced toxicity in rabbits remains insufficient to support standardized recommendations for their routine use.

Future research should focus on prolonged exposure to environmentally relevant OTA concentrations, standardized dose–response studies, sensitive early biomarkers of toxicity, tissue residue kinetics, OTA–pathogen interactions, and rigorous in vivo evaluation of protective strategies. Integration of toxicokinetic, molecular, biochemical, immunological, and pathomorphological endpoints will be essential for improving the assessment of OTA toxicity in rabbits and developing evidence-based preventive and protective strategies. Such approaches may contribute to improved rabbit health and welfare, reduced production losses, and enhanced food-chain safety.

## Figures and Tables

**Table 1:** Clinical, biochemical, hematological, and oxidative alterations associated with OTA exposure in rabbits.

Category	Main Alteration	Toxicological Significance	References
Clinical signs	Reduced feed intake, anorexia, lethargy, depression, dehydration	Early but nonspecific manifestation of systemic OTA toxicity	[30,31,32]
Growth performance	Reduced body weight gain and impaired feed utilization	Reflects chronic metabolic and gastrointestinal impairment	[33]
Renal biomarkers	↑ Urea, ↑ creatinine	Sensitive indicators of progressive renal dysfunction	[26,34,35,36]
Hepatic enzymes	↑ ALT, ↑ AST, ↑ ALP	Evidence of hepatocellular and hepatobiliary injury	[31,34,36,37]
Serum proteins	↓ Total protein, ↓ albumin	May reflect impaired hepatic synthesis, renal protein loss, and reduced nutrient absorption	[34,35,36,37,38]
Glucose metabolism	Usually ↓ glucose, although increases have also been reported	Indicates disturbed carbohydrate metabolism and variable metabolic response	[36,39]
Lipid metabolism	↑ Triglycerides, ↑ cholesterol	Suggests disturbed hepatic lipid metabolism and oxidative injury	[31]
Electrolytes	↓ Chloride; ↓ sodium and potassium in some studies	Reflects systemic metabolic and renal disturbances	[34,35,36]
Erythrogram	↓ RBC, ↓ Hb, ↓ PCV	Consistent with anemia and potential tissue hypoxia	[26]
Leukogram	Lymphopenia, heterophilia/neutrophilia, monocytosis; variable leukocyte responses	Indicates inflammatory activation and immune dysregulation	[36,40]
Hemostasis	↓ Platelets, prolonged coagulation time at high chronic exposure	Suggests hematopoietic and coagulation impairment	[40]
Oxidative stress	↑ ROS, ↑ NO, ↑ MDA, ↑ protein oxidation, ↑ 8-OHdG	Demonstrates lipid, protein, and DNA oxidative injury	[36,41,42]
Antioxidant defense	Altered CAT and SOD; ↓ GPx-1 and antioxidant capacity in advanced toxicity	Indicates depletion or compensatory activation of antioxidant systems	[36,41,42]
Renal injury/fibrosis markers	↑ KIM-1, ↑ COL1A1, ↑ hydroxyproline	Supports proximal tubular injury and progressive fibrotic remodeling	[42,43,44]

**Table 2:** Organ-specific pathological and immunological effects of OTA in rabbits.

Organ/System	Principal Lesions or Functional Changes	Toxicological Interpretation	References
Kidney	Proximal tubular degeneration, vacuolation, epithelial desquamation, tubular dilation, necrosis, glomerular alterations, fibrosis	Primary target organ; progressive nephrotoxicity	[36,41,43,46]
Liver	Granular/vacuolar degeneration, fatty change, hepatocellular necrosis, sinusoidal congestion, biliary proliferation, fibrosis	Major extra-renal target; hepatotoxicity and metabolic dysfunction	[26,31,33,37]
Spleen and thymus	Lymphoid depletion, degeneration/necrosis, reduced white pulp, thymic hypocellularity	Morphological correlate of immunosuppression	[35,47]
Humoral immunity	Reduced antibody response to sRBC and RHDV vaccination	Functional suppression of protective antibody-mediated immunity	[41,47,48]
Cell-mediated immunity	Reduced delayed-type hypersensitivity and lymphocyte proliferation	Suppression of adaptive cellular immunity	[48]
Cytokine response	↑ IL-6, IL-1 β, IFN-γ, TNF-α	Links OTA toxicity with inflammatory signaling and immune dysregulation	[42,49]
Lung	Mild inflammatory lesions after OTA alone; severe bronchopneumonia, croupous pneumonia, oedema, hemorrhage, pleuritis during secondary pasteurellosis	Demonstrates interaction between immunosuppression and infectious disease	[47,50]
Heart	Myofibrillar degeneration, vacuolation, myolysis, necrosis	Evidence of extra-renal systemic toxicity	[36]
Intestine	Villus shortening, epithelial desquamation, lymphocytic infiltration	Impaired nutrient absorption and intestinal barrier integrity	[26,31]
Testes	Spermatogenic epithelial degeneration, seminiferous tubular disruption, oedema, reduced spermatozoa	Male reproductive toxicity	[41]
Fetuses	Reduced fetal growth, skeletal and visceral malformations, hydrocephalus, microphthalmia, renal agenesis	Developmental and teratogenic toxicity	[24,25,26]
Brain/cerebellum	Neuronal pyknosis/lysis, pericellular and pericapillary oedema, microglial proliferation, Purkinje-cell changes	Supports possible neurotoxic involvement	[42]
Secondary infection	Increased *Pasteurella* burden, more severe respiratory disease, increased mortality	Clinically relevant consequence of OTA-induced immunosuppression	[47,50]

**Table 3:** Protective effects of selected phytogenic additives relevant to OTA toxicity in rabbits.

Phytogenic Additive	Principal Protective Properties	Evidence in Rabbits	Effects during OTA Exposure	References
*Silybum marianum*	Antioxidant, hepatoprotective, nephroprotective, immunomodulatory	Improved growth/feed utilization and reproductive performance reported independently of OTA exposure	Reduced severity of OTA-induced renal, hepatic, and other organ lesions; attenuation of adverse effects on body weight	[47]
Silymarin	Antioxidant, hepatoprotective, nephroprotective, immunoprotective	Protective effects on hepatic and clinicopathological parameters demonstrated in rabbits	Pronounced attenuation of OTA-induced suppression of humoral immunity and reduction of renal and hepatic lesions	[47,83,85]
*Withania somnifera*	Antioxidant, anti-inflammatory, hepatoprotective, immunostimulatory	Reduced oxidative stress and improved health-related parameters in rabbits	Marked attenuation of OTA-induced humoral immunosuppression and reduction of renal and hepatic lesions	[47,85]
*Centella asiatica*	Antioxidant, vascular-protective, gastrointestinal-protective, immunomodulatory	Improved growth performance and feed utilization reported in rabbits	Reduced OTA-associated renal and hepatic lesions; immunoprotective effect less pronounced than with silymarin and *W. somnifera*	[47,83]

## Data Availability

No new data were created or analyzed in this study. Data sharing is not applicable to this article.

## Abbreviations

The following abbreviations are used in this manuscript:

OTA	Ochratoxin A
ALT	Alanine aminotransferase
AST	Aspartate aminotransferase
ALP	Alkaline phosphatase
LDH	Lactate dehydrogenase
ROS	Reactive Oxygen Species
MDA	Lipid peroxidation/malondialdehyde
CAT	Catalase
SOD	Superoxide dismutase
NO	Nitric Oxide
8-OHdG	8-hydroxy-2’-deoxyguanosine
COL1A1	Pro-collagen I alpha 1
TNF-α	Tumor necrosis factor alpha
KIM-1	Kidney injury molecule-1
RHDV	Rabbit Haemorrhagic Disease Virus
IL-2	Interleukin-2
IL-6	Interleukin-6
IL-1β	Interleukin-1 beta
IFN-γ	Interferon-gamma
TLC	Thin-layer chromatography
HPLC	High-performance liquid chromatography
HPLC-FLD	High-performance liquid chromatography with fluorescence detection
LC-MS	liquid chromatography–tandem mass spectrometry
ELISA	enzyme-linked immunosorbent assay
